# Effect of pharmacist care on clinical outcomes and therapy optimization in perioperative settings: A systematic review

**DOI:** 10.1093/ajhp/zxae177

**Published:** 2024-06-27

**Authors:** Lina Naseralallah, Somaya Koraysh, May Alasmar, Bodoor Aboujabal

**Affiliations:** Pharmacy Department, Hamad Medical Corporation, Doha, Qatar; Pharmacy Department, Hamad Medical Corporation, Doha, Qatar; Pharmacy Department, Hamad Medical Corporation, Doha, Qatar; Pharmacy Department, Hamad Medical Corporation, Doha, Qatar

**Keywords:** Pharmacist, pharmaceutical care, clinical outcomes, therapeutic outcomes, perioperative settings, surgery

## Abstract

**Purpose:**

Integration of pharmacists into the perioperative practice has the potential to improve patients’ clinical outcomes. The aim of this systematic review is to systematically investigate the evidence on the roles of pharmacists in perioperative settings and the effects of pharmacist interventions on clinical outcomes and therapy optimization.

**Methods:**

A protocol-led (CRD42023460812) systematic review was conducted using search of PubMed, Embase, CINAHL and Google Scholar databases. Studies that investigated the roles and impact of pharmacist-led interventions in the perioperative settings on clinical outcomes were included. Data were extracted and quality assessed independently by two reviewers using the DEPICT-2 (Descriptive Elements of Pharmacist Intervention Characterization Tool) and the Crowe Critical Appraisal Tool (CCAT), respectively. Studies were grouped according to the clinical area into 5 sections: (1) pain control and opioid consumption; (2) venous thromboembolism (VTE); (3) surgery-related gastrointestinal complications; (4) postoperative medication management; and (5) total parenteral nutritional.

**Results:**

Nineteen studies involving a total of 7,168 patients were included; most studies were conducted in gastrointestinal (n = 7) and orthopedics (n = 6) surgical units. Most included studies (n = 14) employed a multicomponent intervention including pharmaceutical care, education, guideline development, drug information services, and recommendations formulation. The processes of developing the implemented interventions and their structures were seldom reported. Positive impacts of pharmacist intervention on clinical outcomes included significant improvement in pain control and reductions in the incidence of VTE, surgery-related stress ulcer, nausea, and vomiting. There is inconsistency in the findings related to medication management (ie, achieving desired therapeutic ranges) and management of chronic conditions (hypertension and type 2 diabetes).

**Conclusion:**

Whilst there is some evidence of positive impacts of pharmacist intervention on clinical outcomes and optimizing drug therapy, this evidence is generally of low quality and insufficient volume. While this review suggests that pharmacists have essential roles in improving the care of patients undergoing surgery, more research with rigorous designs is required.

The perioperative period is defined as the time surrounding the surgical act, which includes 3 phases: preoperative, intraoperative, and postoperative.^1,2^ The perioperative care process is unique and challenging as it occurs in a complex, dynamic, and often time- and resource-constrained environment.^3^ For decades, the process of ensuring safe surgical procedures centered only on the technical aspects; however, recent findings imply that solely enhancing the accessibility of surgical care doesn’t necessarily lead to improved health outcomes unless it was augmented with high-quality perioperative care.^4^ Thus, in recent years, much attention has been devoted to the empowerment of nontechnical skills and interpersonal communication, which contribute to the improvement of patient-oriented priorities such as improving pain control after surgery.^1,5,6^

Perioperative medication management (eg, stopping or continuing medications perioperatively) poses the unique challenge of balancing the risk of complications with the risk of uncontrolled chronic conditions. Studies have shown that a significant number of surgical patients are known to take chronic medications that have been inadequately managed during the perioperative period.^7-9^ Additionally, a prospective survey study assessing the relationship between perioperative medical management and the relative risk of surgical complications concluded that at least 50% of patients undergoing surgeries were receiving chronic medications, and those patients had an increased risk of a postoperative complication (relative risk, 2.7 [95% CI, 1.76-4.04]) when compared to those not receiving chronic medications.^10^ Therefore, perioperative medication management and balancing priorities has been highlighted as a research priority and as a primary goal of perioperative care to prevent or minimize complications, reduce postoperative pain, and accelerate recovery.^6,11^

Recently, multiple efforts have been made to recognize the ever-evolving roles of pharmacists in various settings.^12-15^ Despite the well-defined roles and contributions of most healthcare providers in the perioperative settings, the roles and impact of pharmacist integration into these settings is seldom examined.^16-18^ Owing to its members’ extensive therapeutic knowledge and training, the American Society of Health-System Pharmacists (ASHP) has recognized the paradigm shift in pharmacist involvement in perioperative settings in areas ranging from medication distribution and regulatory compliance to supporting institutional quality and safety goals, developing perioperative treatment algorithms, and collaborating with the surgical team to provide the utmost patient-centered care.^19^ Clinical pharmacists in perioperative units can become an invaluable asset because they possess the capability to systemically assess and analyze patients and their medications through every stage in the surgical journey, thus fulfilling a distinctive role in those units as compared to other inpatient units.^18,20^

This systematic review aims to systematically investigate the evidence on the roles of pharmacists and the effect of pharmacist interventions on clinical outcomes and therapy optimization in perioperative settings. It also will provide insight on areas where future research is required to assist in the formation of pharmacists’ professional identity in surgical settings.

## Methods

In conducting this systematic review, the authors followed the Preferred Reporting Items for Systematic Reviews and Meta-Analyses (PRISMA) guidelines.^21^ The review protocol was registered with the International Prospective Register of Systematic Reviews (PROSPERO) (CRD42023460812).

### Inclusion and exclusion criteria

Studies were included if they were (1) randomized controlled trials (RCTs); quasi-experimental, pre-post, prospective, or retrospective cohort studies; (2) evaluated a clinical pharmacist–led intervention; (3) conducted in the perioperative period; (4) had a control or comparison group (ie, healthcare professionals other than pharmacists); (5) measured any clinical outcome or therapy optimization/appropriateness; and (6) published in a peer-reviewed journal in the English or Arabic languages and available in full-text form. Case reports, expert opinions, systematic reviews, letters to editors, commentaries, correspondences, news articles, and qualitative studies were excluded from this review, as were conference abstracts if not available in full text. We also excluded studies focusing on pediatric patients.

### Search strategy

A search was conducted using PubMed, Embase, and CINAHL for studies published from database inception until September 2023. A search strategy was devised following discussion within the research team. The search strategy was kept deliberately broad to capture all outcomes of pharmacist-led interventions that pertained to medication errors, healthcare utilization, antimicrobial stewardship, and clinical outcomes. In this review, we present the findings related to clinical outcomes and therapy optimization that we were able to retrieve from current literature. A clinical outcome can be defined as measurable changes in symptoms or health status or an event that happens due to an intervention.^22,23^ Therapy optimization involves changes in drug therapy reflecting an increase in appropriateness to enhance patient outcomes.^24^

Keywords and Medical Subjects Headings used in the search comprised 2 categories: pharmacy (‘pharmacist [MeSH]’, ‘pharmacy’, ‘medication therapy management’, ‘pharmaceutical care’, ‘medication counselling’) and perioperative (‘perioperative period [MeSH]’, ‘perioperative care [MeSH]’, ‘surgery’, and ‘procedure’). We combined these keywords using Boolean operators, ie, “OR” and “AND.” Search terms underwent slight modification depending on which database was used. The reference lists of included studies and relevant review articles identified from the search were examined to identify additional publications. In addition, we searched Google Scholar for further studies not identified from the systematic search, whereby the first 500 relevant hits were screened for eligibility.

### Selection process

Titles and abstracts of all retrieved studies were screened independently by two reviewers (L.N. and S.K.). The study team then worked in pairs to screen the full text of identified articles to determine eligibility for inclusion. Any discrepancy or disagreements were initially resolved through discussion in pairs and, if still unresolved, within the extended team. All eligible articles were transferred to Rayyan (Rayyan, Cambridge, MA), a web application for systematic reviews, for duplicates to be removed.^25^

### Data collection process

A bespoke data extraction tool based on the DEPICT-2 (Descriptive Elements of Pharmacist Intervention Characterization Tool) was developed and pilot tested using a sample of the included articles.^26^ The DEPICT-2 is a validated instrument for accurately describing and characterizing the details of pharmacist interventions. The tool consists of 93 items, subsumed into 11 domains: contact with recipient, setting, target population, clinical data sources, variables assessed, pharmacist intervention, timing of intervention, material that support intervention, repetition, communication with recipient, and changes in therapy and laboratory tests.^26^ The final data extraction sheet included the following components:

- General information: author(s), year, country, study design, objectives, population, sample size, study duration, and surgical unit(s)
- Description of intervention: recipients, focus of intervention, setting, method of communication, clinical data source, pharmacist action, timing and frequency of action, and materials that supported action
- Key findings

The included articles were distributed amongst the reviewers (all had expertise in clinical pharmacy), who worked in pairs independently to undertake the data extraction.

### Quality assessment

Quality assessment of the selected articles was undertaken by independent reviewers working in pairs using the validated Crowe Critical Appraisal Tool (CCAT) version 1.4 after a pilot exercise within the research team.^27^ Any discrepancies were resolved through team discussions. The CCAT consists of 22 items grouped into 8 domains and is applicable to all study designs, with the highest possible score being 40. The tool helps in recording scores for each category so that the final score is not influenced by an overall opinion about the study. The quality of studies was categorized by CCAT score as follows: high quality (36 and above), moderate quality (30-35), and low quality (29 and below). This was based on a consensus reached by the reviewers to group studies by quartiles, an approach similar to that adopted by Donnelly et al^28^ and El-Awaisi et al.^29^ The author of the CCAT tool was also contacted to ensure that this method of interpretation was valid.

### Data synthesis

Due to the heterogeneity of study objectives, designs, methods, and outcome measures, a narrative synthesis of the extracted data was undertaken. For the description of pharmacist interventions, the DEPICT-2 was used to summarize the data. For the outcomes of these interventions, studies were grouped and findings presented according to 5 emerging medical conditions themes: pain control and opioid consumption, venous thromboembolism (VTE), surgery-related gastrointestinal complications, postoperative medication management, and total parenteral nutritional (TPN).

## Results

### Study inclusion

**Figure 1.**
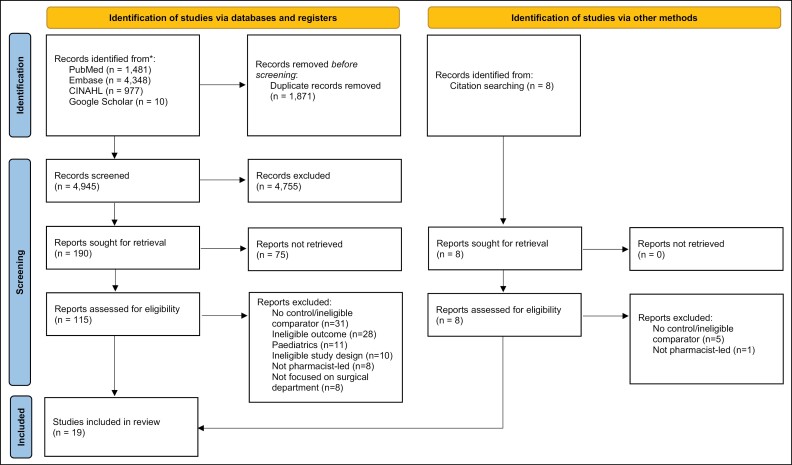
PRISMA flowchart of screening process and reasons for exclusion of studies.

A total of 6,816 records were identified from electronic databases, and 8 records from reference lists of publications. After removal of duplicates, 4,945 records remained for title and abstract screening. A final total of 19 records were included in the review (Figure 1).

### Characteristics of included studies

The characteristics of the included studies are presented in Table 1. Of the 19 included studies, 7 were conducted in China,^36,40,44–48^ 5 in the US,^34,35,37,41,43^ and 1 each in Japan,^42^ Turkey,^39^ Australia,^38^ Canada,^33^ France,^30^ Malta,^31^ and the UK.^32^ The majority of included studies were of pre/post–intervention implementation designs (n = 8),^30,32,33,36,37,45–47^ followed by observational retrospective studies (n = 6)^34,40,41,43,44,48^ and RCTs (n = 4),^31,35,38,42^ with only one observational prospective study.^39^ The studies were conducted at different time intervals and spanned different durations, with the least having a duration over 3 months and the longest a duration over 36 months. A total of 7,168 patients were included in the reviewed studies, which were conducted in surgical wards of gastrointestinal (7 studies),^33,43,45–48^ orthopedics (6 studies),^32,34–37,40^ cardiothoracic (2 studies),^31,42^ and other multiple diverse surgeries (3 studies)^30,41,44^; only Hale et al^38^ did not report the surgical setting of their study.

**Table 1.** Summary of Study Characteristic**s**

Source	Country	Objectives	Study design	Sample size	Surgical unit type	Study duration	Results for main outcome(s)
**Focus of intervention: pain control and opioid consumption**							
Barat et al (2023)^30^	France	Evaluate pain management protocol integrating pharmacy consult	Pre-post quasi-experimental study	250 (125 preintervention, 125 postintervention)	Multiple (orthopedic, odontologic, MF/ENT, digestive and visceral, gynecologic, ophthalmologic, plastic and vascular ambulatory surgery units)	3 months	Difference in pain score on scale of 0-10: mean score in preintervention vs control group, 2.6 vs 1.7; mean difference, −0.9 (95% CI, −1.5 to −0.3); *P* = 0.002
Decelis et al (2015)^31^	Malta	Investigate influence of pharmacist intervention to ease postoperative pain	RCT	100 patients (50 control, 50 intervention)	Cardiothoracic	6 weeks	Mean pain scores decreased significantly in intervention group (*P* < 0.05) Assessment with Pain Analysis Questionnaire, after 6 weeks postoperatively, showed that quality and severity of pain were significantly worse in the control group (*P* values ranged from 0.057 to 0.00) except for tingling and pins and needles–like pain, which did not differ significantly
Fitzpatrick et al (2023)^32^	UK	Investigate significance of pharmacist input, impact on reducing prescribing errors, postoperative patient outcomes, and patient and staff satisfaction with the service	Retrospective pre/post–intervention implementation study	209 patients (80 preintervention, 129 postintervention)	Orthopedic	20 weeks	Proportion of patients requiring oxycodone prescription (9 days follow-up): 38.4% in preintervention group vs 26% in postintervention group; *P* = 0.29
McPhee et al (1991)^33^	Canada	Assess effect of written guidelines and pharmacist-conducted education on prescribing of postoperative narcotics	Prospective interventional study	552 narcotic orders (177 at baseline, 181 in phase 1, 194 in phase 2); 73 patients (29 at baseline, 22 in phase 1, 22 in phase 2)	General (colon surgery, gastroplasty, cholecystectomy)	8 months	Pain control on day 1 (mean [SD] score): ◦ At baseline, 12.4 (6.3) on verbal scale and 60.7 (22.0) on visual scale ◦ In phase 1, 9.4 (6.8) on verbal scale and 53.3 (31.5) on visual scale ◦ In phase 2, 10.0 (7.0) on verbal scale and 50.4 (29.6) on visual scale; not significant Pain control on day 2 (mean [SD] score): ◦ At baseline, 10.5 (7.4) on verbal scale and 47.1 (27.3) on visual scale ◦ In phase 1, 6.4 (6.3) on verbal scale and 39.6 (25.5) on visual scale ◦ In phase 2, 8.7 (8.0) on verbal scale and 36.4 (29.3) on visual scale; not significant Appropriate dose (% of orders): baseline, 82%; phase 1, 88%; phase 2, 93%; not significant Appropriate frequency (% of orders): baseline, 67%; phase 1, 88%, phase 2, 90%; not significant
Patel et al (2022)^34^	US	Evaluate effect of standardized postoperative multimodal pain regimen guidance on cumulative opioid prescribed postoperatively at discharge, as measured by morphine usage	Retrospective observational study	92 patients (40 pre intervention, 52 post intervention)	Orthopedic	8 months	Cumulative MED at discharge per patient: 560.0 vs 300.0; *P* < 0.001 Cumulative opioid pills: 70.0 vs 60.0; *P* = 0.03 MED per prescription: 350.0 vs 150.0; *P* < 0.001 Opioid pills per prescription: 44.5 vs 30.0; *P* < 0.001 MEDD: 60.0 vs 50.0; *P* < 0.001 Proportion of patients prescribed ≥50 MEDD: 82.5% vs 53.8%; *P* = 0.004 Inpatient postoperative pain scores: 4.0 vs 5.0; *P* = 0.63 Proportion of patients dispensed at least one subsequent opioid prescription: 60% vs 51.9%; *P* = 0.44 Dispensed opioid prescriptions: 3.0 vs 2.0; *P* = 0.92 Cumulative MED: 790.5 vs 450.0; *P* = 0.23 MED per prescription: 269.6 vs 242.3; *P* = 0.12 MEDD: 28.5 vs 21.4; *P* = 0.15 Pain scores from discharge to 14 days post discharge: 5.0 vs 4.3; *P* = 0.90 14 days post discharge to 1 month post discharge: 5.0 vs 2.8; *P* = 0.13
Smith et al (2018)^35^	US	Determine whether pharmacist-led, patient-directed intervention reduces opioid use following total hip arthroplasty or total knee arthroplasty	Randomized, pragmatic clinical trial	561 patients (286 usual care, 275 intervention)	Orthopedic	NR	Total dispensed morphine equivalents (overall), mean (95% CI): 7.22 (7.02-7.41) with usual care vs 7.04 (6.82-7.27) with intervention; crude difference, –0.17 (–0.47 to 0.12) Count of opioid dispensings, mean (95% CI): 3.72 (3.39-4.05) with usual care vs 3.43 (3.10-3.75) with intervention; crude difference, –0.29 (–0.76 to 0.17),
Wang et al (2023)^36^	China	Construct a perioperative pharmaceutical care model pathway for pain management assessment	Before-after study	320 patients (158 control, 162 intervention)	Orthopedic	1 year	Pain at rest 24 hours after surgery: mean score, 3.2 in control group vs 2.4 with intervention; *P* < 0.001) Movement-evoked pain: mean score in control vs intervention group 24 hours after surgery, 4.5 vs 3.1 (*P* < 0.001); on day 2, 3.3 vs 2.6 (*P* < 0.001); on day 3, 2.8 vs 2.5 (*P* < 0.001) Hospital stay in patients with expected severe postoperative pain: mean, 13.6 days in control group vs 10.2 days in intervention group (*P* = 0.001) Breakthrough pain: 22.8% of control group vs 21% of intervention group; *P* = 0.155)
**Focus of intervention: VTE prophylaxis**							
Cronin et al (2009)^37^	US	Asses the effect of a multidisciplinary, pharmacy-led, thromboprophylaxis program on the rates of VTE in total joint arthroplasty patients	Before-after study	1956 patients (953 before intervention, 1,003 after intervention)	Orthopedic	NR	Rate of any VTE: 4.6% before intervention vs 2.4% after intervention (48% reduction) Rate of pulmonary embolism: 0.9% before intervention vs 0.4% after intervention (57% reduction)
Hale et al (2013)^38^	Australia	Evaluate a new model of service for inpatient medication prescribing by a pharmacist in an elective surgery preadmission clinic (PAC) against usual care	RCT	384 patients (190 control, 194 intervention)	NR	3 months	Rate of appropriate VTE prophylaxis: 90% in controls vs 93% with intervention; *P* = 0.29
Kiraci et al (2023)^39^	Turkey	Evaluate VTE risk and prophylaxis appropriateness in hospitalized patients in general surgery wards and examine improvement with CP education	Prospective study	800 patients (340 in pre-education period, 269 in posteducation period, 191 in CP intervention period)	Multiple	10 months	Pre-education vs posteducation vs CP intervention period: VTE incidence, 1.2% vs 0.4% vs 0% Major postoperative bleeding, 1.8% vs 1.9% vs 1.0% Optimal thromboprophylaxis rate in pre-education vs post-education period, 40.6% vs 45.4% (*P* = 0.238); in pre-education vs CP intervention period, 59.2%; *P* = 0.004
Shang et al (2021)^40^	China	Assess impact of clinical pharmacist services on use of anticoagulant drugs, rationality of medication, and incidence of thrombosis in patients undergoing total joint arthroplasty	Retrospective observational study	577 patients (240 baseline, 377 intervention)	Orthopedic	36 months	Rate of VTE prevention (baseline vs intervention): ◦ Preoperative (24.17% vs 37.09%; *P* = 0.001); preoperative of femoral neck fractures (53.77 vs 84.62%; *P* = 0.000) ◦ Postoperative (97.08% vs 98.81%; *P* = 0.040) Medication selection (baseline vs intervention): ◦ Rivaroxaban (0% vs 32.34%; *P* = 0.000) ◦ Aspirin (6.25% vs 39.47%; *P* = 0.000) ◦ LMWH (97.08% vs 98.52%; *P* = 0.234) ◦ VKA (0.83% vs 0.59%; *P* = 0.732) Timing of administration (baseline vs intervention): ◦ ≤24 hours (35.83% vs 81.31%; *P* = 0.000); >24 hours (64.17% vs 18.69%) Course of treatment (baseline vs intervention): ◦ <10 days (27.5% vs 17.5%; *P* = 0.004); ≥10 days (72.5% vs 82.49%)
**Focus of intervention: postoperative acute and chronic medication management**							
Alsheikh et al (2021)^41^	US	Evaluate impact of clinical transplant pharmacy services on kidney transplant program following introduction of these services in terms of inpatient length of stay and all-cause 30-day readmission rates	Retrospective cohort study	205 patients (101 preintervention, 104 postintervention)	Transplant	2 years	Mean (SD) values in preintervention vs postintervention group: Serum creatinine (mg/dL) 1 month post transplant, 2.15 (2.09) vs 2.11 (1.34); 95% CI, −0.444 to 0.526 (*P* = 0.864) Tacrolimus concentration (ng/mL) at POD 7, 7.15 (3.30) vs 6.95 (3.15); 95% CI, −0.719 to 0.111 (*P* = 0.673) Tacrolimus concentration (ng/mL) 1 month post transplant, 8.48 (2.85) vs 8.62 (2.52); 95% CI, −0.896 to 0.608 (*P* = 0.706) Mycophenolate daily dose (mg) at POD 7, 1,886.14 (281.79) vs 1,888.35 (296.48); 95% CI, −82.092 to 77.671 (*P* = 0.957) Mycophenolate daily dose (mg) at 1 month post transplant, 1,764.85 (349.68) vs 1,815.53 (363.73); 95% CI, −149.215 to 47.850 (*P* = 0.311) No. (%) of patients in preintervention vs postintervention group: Delayed graft dysfunction, 46.5% vs 46.1%; 95% CI, 0.569-1.705 (*P* = 0.956) CMS pretransplant pharmacist note: 80.2% vs 95.1%; 95% CI, 1.758-13.599 (*P* = 0.001) CMS posttransplant pharmacist note, 66.3% vs 99.0%; 95% CI, 6.988-390.96 (*P* < 0.001) CMS discharge pharmacist note, 12.8% vs 87.5%; 95% CI, 20.813-107.879 (*P* < 0.001)
Katada et al (2017)^42^	Japan	Evaluate efficacy of protocol-based pharmacotherapy management in warfarin therapy by comparing to conventional pharmaceutical care	RCT	145 patients (77 control, 68 intervention)	Cardiothoracic	2 years	Time in therapeutic range (%), mean (SD): 47.1 (18.7) vs 34.4 (26.8); *P* = 0.002 Time below range (%), mean (SD): 51.7 (18.9) vs 63.2 (27.7); *P* = 0.005 Time above range (%), mean (SD): 1.0 (3.9) vs 2.3 (7.3); *P* = 0.203 Days to reach steady state, mean (SD): 7.3 (3.0) vs 2.3 (7.3); *P* = 0.034
Smith et al (2023)^43^	US	Evaluate differences in mean blood glucose levels, glucose values within goal range, and postoperative outcomes with clinical pharmacist–driven glycemic control vs standard care	Retrospective observational study	186 patients (66 standard care, 120 intervention)	Colorectal	16 months	Postoperative mean blood glucose, mean (SD): 148.3 (44.2) vs 133.9 (34.2); *P* < 0.001 Incidence of hyperglycemic events: 20.5% vs 9.6%; *P* < 0.00001 Ratio of blood glucose values within the goal range: 71% vs 83.4%; *P* < 0.0001 Rate of hypoglycemia events: 0.7% vs 1.2%; *P* = 0.1443
Yang et al (2019)^44^	China	Comprehensive assessment of impact of pharmacist-led posttransplant medication management for kidney transplant recipients	Retrospective cohort study	204 patients (84 preintervention, 120 postintervention)	Transplant	2 years	Percentage of tacrolimus level within normal range at postoperative day 3 (52.38% vs 61.67%; *P* = 0.186) and day 7 (75.00% vs 87.50%; *P* = 0.021) Mean (SD) SBP (141.55 [14.62] mm Hg vs 136.04 [13.17] mm Hg; *P* = 0.01) and BP control rate (67.50% vs 90.70%; *P* = 0.00) Mean (SD) plasma glucose (5.54 [1.31] vs 5.27 [2.42]; *P* = 0.12) and plasma glucose control rate (80.00% vs 96.00%; *P* = 0.13)
**Focuses of intervention: surgery-related stress ulcer and nausea/vomiting prophylaxis**							
Luo et al (2017)^45^	China	Evaluate impact and cost vs benefit of clinical pharmacist interventions on inappropriate use of prophylactic acid suppressant	Retro-prospective intervention study	448 patients (218 preintervention, 230 postintervention)	Hepatobiliary	3 months	Rate of correct acid suppressant administration in surgical patients for stress ulcer prophylaxis: 17.59% vs 27.22%; *P* = 0.023 Adherence to 5 criteria of acid suppressant administration (indication, choice, dose, route, and duration): 0% vs 10.65%; *P* < 0.001
Wang et al (2020)^46^	China	Evaluate effects of clinical pharmacist–led guidance team on the improvement of PONV and prophylaxis administration.	Prospective before-after study	156 patients (82 preintervention; 74 postintervention)	Multiple (abdominal surgeries)	1 year	PONV: 56.10% vs 33.78% (OR, 0.29; 95% CI 0.13-0.64; *P* = 0.002) Vomiting within 24 hours of surgery: 45.12% vs 29.73%, (OR, 0.42; 95% CI, 0.20-0.90; *P* = 0.026) Nausea within 24 hours of surgery: 56.1% vs 31.08% (OR, 0.26; 95% CI, 0.12-0.57; *P* = 0.001) Persistance of PONV improvement post intervention after discharge: 1st month (33.33%) vs 4th month (27.27%); *P* = 0.651 Physician administration of prophylactic antiemetic: OR, 5.82; 95% CI, 2.32-14.59; *P* < 0.001 Accurate timing of prophylactic therapy: OR, 3.66; 95% CI, 1.55-8.66; *P* = 0.003
**Focus of intervention: total parenteral nutrition**							
Tong et al (2022)^47^	China	Evaluate role of pharmacists in managing postoperative complications and nutritional status of perioperative patients with colorectal cancer	Retrospective pre-post cohort study	284 patients (137 preintervention, 147 postintervention)	Colorectal	NR	Proportion of TPN with NPE at 20-35 kcal/kg/day: 60.6% vs 70.7%; *P* = 0.004 Ratio of energy from fat to energy from carbohydrates, up to 50%: 96.4% vs 100%; *P* > 0.05) Proportion of patients with serum albumin decrease: 76.7% vs 25.2% (*P* < 0.05); mean (SD) decrease, −8.11 (0.43) g/L vs −3.08 (0.44) g/L (*P* < 0.0001) Postoperative infection rate: 18.2% vs 11.6% Mean (SD) infection time: 9.4 (1.4) days vs 7.7 (1.0) days; *P* = 0.368 Incidence of TPN-related hypoglycemia: 15.3% vs 5.4%
Wang et al (2019)^48^	China	Evaluate the effect of computerized total parenteral nutrition standardization led by pharmacist in surgical department of abdominal oncology at cancer hospital	Retrospective observational study	218 patients (121 control, 97 intervention)	Colorectal	6 months	Incidence of unreasonable lipid ratio: 25.7% vs 2.1%; *P* < 0.01 Incidence of unreasonable thermal nitrogen ratio: 38% vs 3.1%; *P* < 0.01) Rational use of alanyl glutamine: 90.9% vs 100%; *P* < 0.05 Rate of qualified total TPN prescriptions: 47.1% vs 88.7%; *P* < 0.001

Abbreviations: LMWH, low-molecular-weight heparin; MED, morphine equivalentn dose; MEDD, morphine equivalent daily dose; MF/ENT, maxillofacial and ear, nose, and throat; NPE, nonprotein energy; NR, not reported; OR, odds ratio; POD, postoperative day; SBP, systolic blood pressure; TPN, total parenteral nutrition; VKA, vitamin K antagonist.

### Quality assessment

**Figure 2.**
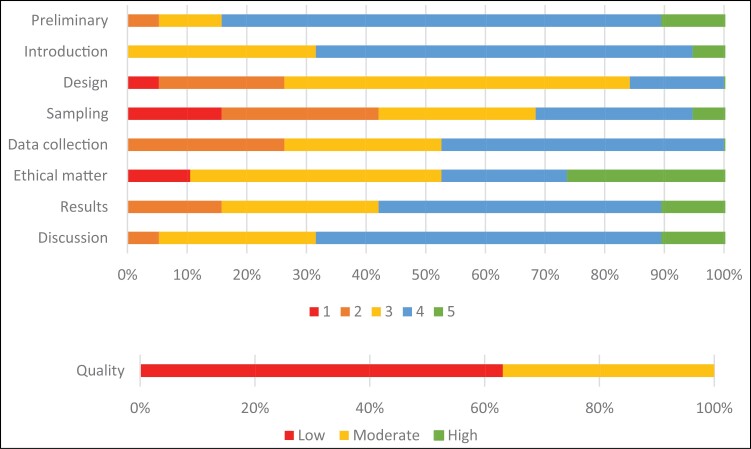
Stacked bar chart representing quality ratings of included studies.

Total CCAT scores ranged between 16 and 32, with a median score of 27.53. The majority of studies (n = 12) were of low quality, while the reminder of the studies (n = 7) were of moderate quality. None of the included studies was of high quality. The lowest average scores were mostly due to deficiencies in sampling, followed by deficiencies in design and data collection (Figure 2, Appendix A).

### Characteristics of pharmacist interventions

**Figure 3.**
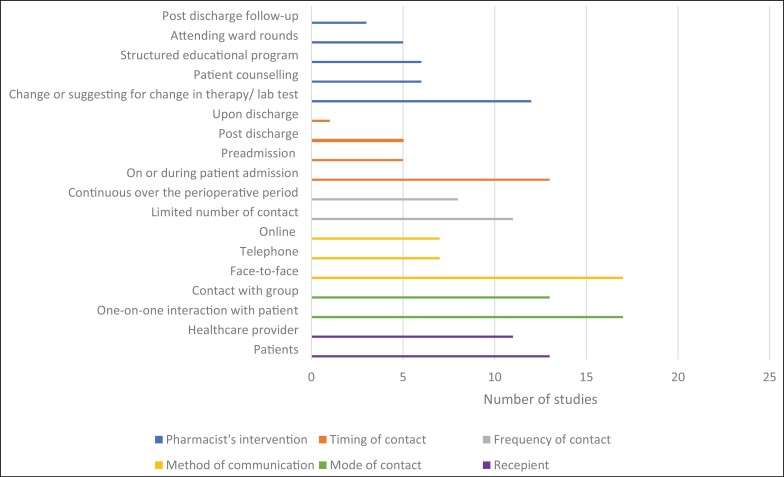
Summary of characteristics of pharmacist-led interventions, as determined by DEPICT-2 assessment.

Figure 3 and Appendix B outline the key characteristics of the pharmacist interventions, as determined by DEPICT-2 assessment.^26^ Recipients of pharmacist-led interventions varied across the studies, with 8 studies reporting on patient-directed interventions,^30–32,35,37,43,44,48^ 8 studies on healthcare professional–directed interventions,^33,34,39,42,45,47^ and 5 studies directed towards both healthcare professionals and patients simultaneously,^36,38,40,41,46^ Furthermore, communication with intervention recipients was either through face-to-face communication (n = 10),^30,31,34,37,38,40,42–45^ or a mixture of different method, including face-to-face, written, and online communication methods (n = 7),^32,33,35,36,41,46,47^ while only 2 studies had no clearly described intervention communication.^39,48^ Only 8 of the included studies incorporated continuous follow-up with patients during their admission period^37,40,42–46,49^ while the reminder of the studies reported a limited number of contacts during perioperative care.^30–33,35,36,38^ Two studies did not report on the frequency of contact.^34,39^

Most studies (n = 14) examined multicomponent interventions.^30,32–41,44–46^ The key intervention components described were pharmaceutical care (development of an individualized pharmaceutical care plan during admission and on discharge, management of adverse effects and drug interactions, medication reconciliation, and patient education), educational programs, guideline development, drug information services, and recommendations formulation (Figure 3, Appendix B). One study also included an independent prescribing element with physician countersignature.^38^ Single-component intervention was investigated in 5 studies.^31,42,43,47,48^ Three of these studies were based on education of staff, guidelines, and medication review,^31,42,43^ with 2 studies exploring a pharmacist-developed decision support system for TPN orders.^47,48^

All included studies lacked information in relation to intervention development. Studies that included some information only reported that local or international clinical practice guidelines were utilized as the basis for the scientific factor of the intervention, without any further details regarding the structures and processes.^31,32,34,36–40,42,43,45–47,49^ Smith et al^35^ reported that the brochures were developed by the research team using qualitative methods along with input from patients and orthopedic surgeons, while the telephone calls were based on the motivational enhancement principles.

### Effect of pharmacist interventions on outcomes measured

 The final column of Table 1 provides a detailed summary of the main results and statistical significance values (if reported) related to each of the studies summarized below. Retrieved studies investigated the impact of pharmacists’ interventions in perioperative settings on diverse clinical outcomes and therapeutic optimization. We categorized our synthesis based on the medical condition of interest into 5 sections: (1) pain control and opioid consumption; (2) venous thromboembolism (VTE); (3) surgery-related gastrointestinal complications; (4) postoperative medication management; and (5) total parenteral nutrition (TPN).

#### Pain control and opioid consumption

A total of 7 studies reported on pain- and opioid-related outcomes; 3 of them reported on pain control,^^30,31,36^^ 3 on opioid consumption,^^32,34,35^^ and one on both^^33^^. Pain control was measured across the studies using different approaches and scales, and outcomes were reported as either pain scores or mean differences in pain between arms. Wang et al^36^ demonstrated a reduction in scores for pain (both at rest and during movement) 24 hours post orthopedic surgery (*P* < 0.001); however, there was no significant difference in breakthrough pain. Barat et al^30^ focused on multiple surgical units and reported a mean difference in pain control scores of –0.9 (95% CI, −1.5/10; −0.3/10; *P* = 0.002).^30^ Similarly, Decelis et al^31^ conducted an RCT on patients undergoing cardiothoracic surgery and measured pain outcomes utilizing the Pain Analysis Questionnaire at 6 weeks post surgery. The findings showed that the quality and severity of pain were significantly worse in control group patients, with the exception of tingling and pins and needles–like pain.^31^

Opioid consumption across the studies was measured as dispenses or dosages of morphine equivalents and/or opioid prescriptions. Smith et al^35^ and Patel et al^32^ showed, respectively, reductions in the cumulative dispensed morphine equivalents and cumulative morphine dosages on discharge in orthopedic patients. Similarly, Fitzpatrick et al,^32^ who also focused on orthopedic patients, reported a reduction in required oxycodone prescriptions. Finally, McPhee et al,^33^ who studied both outcomes, reported more appropriate use of opioid prescriptions in the pharmacist intervention arm; however, no significant difference was noted in pain control across their 2-phase study (phase 1, guideline development; phase 2, educational program).

#### Perioperative VTE prophylaxis

Four studies reported outcomes related to the impact of perioperative pharmacist services on VTE prophylaxis^37–40^ Three studies showed that pharmacist-led interventions resulted in an increased rate of appropriate administration of pharmacologic prophylaxis for VTE.^38–40^ Additionally, Shang et al^40^ reported a significant increase in the timely administration of VTE prophylaxis after orthopedic surgery as well as increased utilization of rivaroxaban and aspirin, with no significant change in the trends of low-molecular-weight heparin and vitamin K antagonist use, after implementation of pharmacist intervention.

Two studies also reported that pharmacist interventions led to a reduction in VTE incidence; however, statistical significance was not reported in either study.^37,39^ In fact, Kiraci et al^37^ reported a reduction in the frequency of VTE events from 1.2% to 0% after implementing a clinical pharmacist, and the follow-up period was extended to up to 30 days.^39^ Cronin et al^37^ focused on patients undergoing total joint arthroplasty and showed 48% and 57% reductions in the incidence of any VTE and the incidence of pulmonary embolism, respectively.

#### Postoperative medication management

*Acute management.* Management of acute medications added and necessitated after surgical intervention were examined by two studies in renal transplant wards^41,44^ and one study in a cardiothoracic ward.^42^ Yang et al^44^ and Alsheikh et al^41^ reported outcomes on therapeutic immunosuppressive drug management post transplant. Yang et al reported a significantly higher incidence of attaining a normal tacrolimus level after pharmacy intervention at postoperative day 7 (87%, compared to 75% without pharmacist intervention; *P* = 0.021).^44^ In contrast, a US-based retrospective cohort study showed that pharmacist involvement was not associated with improvements in tacrolimus concentrations and mycophenolate dosages at day 7 and at 1 month post surgery.^41^

A Japan-based RCT that focused on patients undergoing cardiothoracic surgeries investigated postoperative warfarin anticoagulation outcomes.^42^ Findings showed that pharmacist intervention resulted in an increased likelihood of adequate time in the therapeutic range (*P* = 0.002) and decreased time in both supra- and subtherapeutic ranges (*P* = 0.203 and *P* = 0.005, respectively).

##### Chronic comorbidities management

Two studies explored the impact of pharmacist intervention on the management of chronic comorbidities postoperatively; the only endpoints investigated were blood pressure (BP) and blood glucose (BG).^43,44^ Smith et al^43^ evaluated the impact of clinical pharmacist–driven glycemic management as compared to standard care in patients undergoing colorecta surgeries. Their findings showed statistically significant improvements in the postoperative mean BG levels, hyperglycemic events, and ratio of BG values within the goal range, with no increase in the rates of hypoglycemia events. However, Yang et al^44^ reported no difference in glycemic control between the pharmacy intervention and control groups; it is noteworthy that glycemic control was investigated as a secondary outcome in that study.

Only one study reported on the impact of pharmacist services on BP control; significant improvements were noted in both systolic BP and BP control rates in the postintervention group versus the preintervention group.^44^

#### Surgery-related gastrointestinal complications

Two studies reported on gastrointestinal complications post surgery, including stress ulcer^45^ and nausea and vomiting.^46^ A China-based study that included patients undergoing hepatobiliary surgeries reported that prescriber adherence to the 5 criteria of appropriate administration of acid-suppressive medication (proper indication, choice, dose, route, and duration) significantly increased, by 10.65% (*P* < 0.001), with pharmacist intervention.^45^ An increased rate of the appropriate use of proton pump inhibitors (PPIs) for stress ulcer prophylaxis was also observed.^45^

A positive impact of pharmacist intervention on postoperative nausea and vomiting (PONV) was reported by Wang et al,^46^ who demonstrated a substantial reduction in PONV incidence with pharmacist-provided services, with a calculated number needed to treat of 4 patients; statistical significance held when nausea and vomiting were assessed as individual outcomes. Additionally, the study evaluated rates of persistent nausea and vomiting after discharge and showed similar rates in both groups. Due to pharmacist intervention, there was a significant increase in the administration of prophylactic antiemetics and an increase in the timely administration of antiemetics.^46^

#### TPN management

Two studies explored the impact of pharmacist-developed evaluation standards and TPN software on the appropriateness of TPN prescribing and clinical outcomes.^47,48^ Wang et al^48^ reported an 88.7% rate of TPN prescription optimization with pharmacist input, compared to a rate of 47.1% with standard care. Additionally, significant reductions in “unreasonable” lipid ratios and thermal nitrogen ratios were observed.^48^ Both studies showed a decrease in postoperative prealbumin and albumin levels with pharmacy-integrated TPN prescriptions. Furthermore, Tong et al^47^ investigated clinical outcomes such as infection rates and duration and TPN-related hypoglycemia, but none of the findings were were statistically significant.

## Discussion

### Main findings

Pharmacist-led interventions appear to positively impact clinical outcomes and drug therapy optimization in the surgical setting; however, the available evidence is of low quality and insufficient volume. In the reviewed studies, pharmacist interventions resulted in a reduction in opioid consumption while improving pain control among surgical patients. Pharmacist involvement also led to improvement in the proper use of VTE prophylaxis, which subsequently resulted in reduced occurrence of VTE. Findings from the current review also suggest that pharmacist interventions markedly enhance adherence to the proper administration of acid-suppressive therapy and significantly decrease PONV, which reduces gastrointestinal complications following surgery. Pharmacists were found to play a role in optimizing TPN prescriptions, but the clinical significance of this role is yet to be delineated. Additionally, there is inconsistency in the findings related to medication management, specifically concerning immunosuppressive, hypertension, and antihyperglycemic medications.

Most included studies employed a multifaceted intervention including pharmaceutical care (development of an individualized pharmaceutical care plan during admission and on discharge, management of adverse effects and drug interactions, medication reconciliation, patient counseling), education, guideline development, drug information services, and providing recommendations. There is lack of data on the processes and structures of developing interventions among the included studies, with all reviewed studies only commenting on the basis of the scientific component of the interventions.

### Findings in the context of other research

The outcomes of this review pertain exclusively to the surgical context, characterized by its distinct nature, as numerous interventions take place throughout the surgical process. This uniqueness necessitates frequent manipulation of medications, transitioning between formulations, and occasionally resorting to alternative approaches.^50^

#### Pain management

Numerous studies emphasized the involvement of pharmacists in pain management in a variety of settings, with the majority of the substantial evidence primarily focusing on the management of chronic pain.^51-55^ A meta-analysis of 12 RCTs was undertaken to explore the influence of pharmacist involvement on the intensity of pain.^52^ The results of that investigation revealed that pharmacist interventions led to a statistically significant decrease in pain intensity relative to outcomes in the control group. Notably, the effectiveness of the intervention was heightened when a pharmacist administered a comprehensive set of services encompassing educational interventions, medication review, and pharmaceutical care services. However, no statistically significant difference was observed when considering educational interventions in isolation.^52^ Another retrospective cohort investigation assessed the impact of pharmacist pain consultation services within the inpatient setting.^53^ The study revealed a statistically significant reduction in the average pain score, measured on a scale of 0 to 10, from the preconsult to the postconsult period (6.15 vs 3.25; *P* < 0.001). Furthermore, the achieved pain relief was consistently maintained throughout the entire hospital stay (mean predischarge pain score, 3.6; *P* < 0.001).^53^ Research supports the conclusions drawn in our review, indicating that pharmacists have a crucial role in managing acute pain. It also is noteworthy that our findings showed that pharmacist interventions reduced the consumption of opioids while improving pain control, which is usually conceived as a dilemma in perioperative settings.^54^ Pain management in surgical patients presents a more significant challenge than in other types of patients, underscoring the potential for further exploration of the role of pharmacists in future research.^55^

#### VTE prevention

In a meta-analysis conducted in the outpatient setting, pharmacist-led anticoagulation management resulted in fewer total hemorrhagic events compared to other management models.^56^ However, the findings regarding thrombotic events were conflicting, as pharmacist intervention led to a reduction in thrombotic events in observational studies but no significant difference in RCTs.^56^ One prospective observational cohort study investigated the influence of pharmacists on a 6-month composite endpoint encompassing complications related to anticoagulation treatment. Each study arm had a single occurrence of the composite primary endpoint (an incidence rate of 6%), and both instances were attributed to recurrent VTE events.^57^ In another study, a pharmacist-led initiative targeting medical patients to enhance thromboprophylaxis strategies was implemented. Following the campaign, there was a notable increase in the rate of thromboprophylaxis ordering for high-risk patients, which rose from 15.2% to 43.1%.^58^ The findings outlined above underscore crucial considerations, with the foremost being the need for further research specifically crafted to identify VTE events. This necessity arises from the observation that VTE rates generally fall below 1% across a majority of surgical procedures, thereby demanding extensive sample sizes and prolonged study durations in order to draw definitive conclusions regarding the influence of pharmacists on reducing VTE rates.^59^ Furthermore, there is a need for additional research that examines the broader implications of pharmacist intervention in anticoagulation, extending beyond a focus solely on VTE prophylaxis. This research should explore the involvement of pharmacists in the care of patients undergoing chronic anticoagulation therapy, particularly in surgical settings where interruptions in chronic anticoagulation therapy are often necessary. It is essential to conduct studies to assess the pharmacist’s role in clinical decision-making within this specific context.

#### Management of chronic conditions

Pharmacists perform distinctive and crucial functions in improving the management of multiple chronic conditions, including hypertension. In a review conducted by El-Awaisi et al,^23^ the impact of interventions led by pharmacists in Arab countries on various patient outcomes was examined. The systematic review revealed a notable reduction in BP readings across all studies incorporating pharmacist-led interventions. It is noteworthy that all the studies involved an extended follow-up duration of at least 3 months. Chonko et al^60^ showed that pharmacists effectively oversaw the hypertension of patients in an ambulatory care setting, leading to an average reduction in systolic BP exceeding 10 mm Hg (*P* = 0.0035) in pharmacist-managed patients compared to those without pharmacist management. The studies included in the review by El-Awaisi et al^23^ and the study by Chonko et al^60^ involved a smaller sample size than the studies on BP control incorporated in this review. Additionally, those studies featured multiple visits with a pharmacist spanning 1 year, unlike those included in our review, in which there were limited points of contact with patients and no follow-up. Existing literature suggests that achieving optimal BP readings may necessitate medication adjustments over a period of up to 7 months,^61^ but notably, the supporting evidence is from a review focused on a patient population of kidney transplant recipients. This group faces more constrained options for managing hypertension and is predisposed to persistently elevated BP readings relative to individuals without this specific comorbidity.^62^

**Key Points**

- Pharmacist-led interventions appear to positively impact clinical and therapeutic outcomes in the surgical setting; however, the available evidence is of low quality and insufficient volume.
- Complex and multicomponent pharmacist interventions that span the whole perioperative journey are more likely to yield positive effects.
- There is a lack of data on the development of pharmacist-led interventions in terms of structure and processes, which might hinder the reproducibility of these interventions.

The systematic review of El-Awaisi et al^23^ investigated pharmacist intervention in various settings, encompassing both community and hospital environments. The pharmacist-led interventions resulted in a decrease in glycated hemoglobin, fasting blood glucose, and postprandial blood glucose. Notably, the studies included in that review featured extended follow-up periods.^23^ In an investigation led by Mularski et al,^63^ a team of pharmacists known as the Glycemic Control Team (GCT) was implemented. The study revealed a substantial increase in good glycemic control on day 1 after intervention by the GCT, with an odds ratio of 3.10 (*P* < 0.0001). Concurrently, there was a noteworthy decrease in the occurrence of hypoglycemia from 8.6% to 4.6% (*P* < 0.0001) during the corresponding timeframe. These findings diverge from the outcomes observed in the studies reviewed in this article. It is essential to highlight that, in contrast to the 2 pertinent studies incorporated in our review, the primary focus of the study by Mularski et al was diabetic outcomes. Additionally, that study encompassed a substantial sample size of over 5,000 patients, resulting in high statistical power for assessing diabetic outcomes.^63^ Another study highlighted that pharmacist-driven glycemic control significantly reduced severe hypoglycemia in high-risk patients.^64^

#### Gastrointestinal complications

In a nonrandomized controlled study carried out in primary care settings, Wong et al^65^ reported a substantial reduction in the rate of inappropriate PPI utilization (from 79.9% to 30.4%; *P* < 0.05) within the pharmacist intervention group compared to the control group. In a prospective interventional study within a general surgery department,^66^ a pharmacist intervention group had a markedly increased overall rate of appropriate PPI utilization when compared to a control group (*P* < 0.01). Furthermore, there were significant reductions in unjustified PPI use, lower PPI utilization rates, decreased drug costs, and diminished PPI-specific costs in the intervention group versus the control group (*P* < 0.05 for all between-group comparisons).^66^ Similar findings were reported in a study of pharmacist intervention in PPI usage in the ICU setting.^67^ These studies reaffirm the role emphasized in our review, elucidating the advantages of interventions led by pharmacists aimed at encouraging the appropriate utilization of PPIs.

Only a few studies have evaluated the role of pharmacists in nausea and vomiting prevention in surgical settings. In a study assessing the effects of a pharmacist-led initiative targeting refractory chemotherapy-induced nausea and vomiting (CINV), Quinn et al^68^ demonstrated that due to pharmacist intervention, 89.1% of patients experienced a collective decrease from baseline in both nausea and vomiting. In another retrospective investigation, pharmacists conducted a phone consultation intervention for patients receiving emetogenic chemotherapy.^69^ The primary composite endpoint, encompassing emergency department visits, hospital admissions, and infusion center appointments related to CINV within 30 days of moderately or highly emetogenic chemotherapy, exhibited an incidence of 1.5% in the intervention group as opposed to 7% in the control group (*P* = 0.129).^69^ These results indicate that the contribution of pharmacists to the mitigation of nausea and vomiting remains consistent across various settings. Nevertheless, it is noteworthy that the approaches employed in these studies differed from those used in the studies reviewed here. Many of those studies incorporated an educational component directed at patients concerning nausea and vomiting, antiemetics, and alternative approaches for managing these symptoms. Importantly, the absence of such a patient education element in the studies included in this review highlights the potential for alternative pharmacist-led approaches in PONV.

### Characteristics of pharmacist interventions

Most of the previous studies in this area encompassed various services within the field of clinical pharmacy practice. These services included admission reconciliation, medication review, communication with prescribers to optimize medication, monitoring, and patient education.^70,71^ Some studies focused on unique educational initiatives directed toward healthcare providers or patients. The interventions in these studies were multifaceted, allowing proactive engagement at different care stages. Existing research has supported the notion that care transitions, particularly during discharge or transfer, significantly contribute to potential gaps in practice, such as not resuming medications or not prescribing needed medications.^72^ Within the perioperative setting, the number of transitions far exceeds that of other care domains. Patients undergo numerous transitions and changes in location and healthcare providers quickly, necessitating dynamic and diverse pharmacist interventions to address these complexities effectively.^73^ Several articles included in our review implemented pharmacist interventions with limited contact frequencies, typically restricted to 1 or 2 contact points within the process. This limitation in contacts may lead to substantial underestimation of the impact of pharmacists’ role, as many instances of potential intervention will be missed.

Notably, only a minority of the studies in the review investigated the utilization of pharmacy prescribing services, specifically independent pharmacist prescribers (IPPs). A comprehensive cross-sectional study demonstrated that IPPs had an error rate of 0.7% (95% CI, 0.0%-1.0%), contrasting with physicians, who exhibited a substantially higher error rate of 9.8% (95% CI, 9.0%-11.0%).^74^ Additionally, the reviewed studies needed to report on the pharmacist-to-patient ratio. This deficiency in pharmacist staffing is particularly evident in surgical settings, where pharmacists often handle a higher patient load than their counterparts in medical or intensive care unit (ICU) settings, potentially leading to an underestimation of the positive role of pharmacy.^18^

A notable portion of the included studies employed a pragmatic approach in implementing pharmacist interventions, predominantly relying on international, national, or institutional therapeutic guidelines as the scientific basis for interventions. Importantly, there is a substantial deficiency in the execution and documentation of the processes involved in developing and adapting pharmacist interventions within the current settings. This shortfall extends to clarifying the rationale for selecting each element constituting the intervention and the scientific expectations regarding its impact on outcomes.^75^ Improved understanding in these areas could enhance participant engagement in the studies and bolster the generalizability and reproducibility of the research findings.^76,77^ The absence of reporting the theoretical foundations of the interventions limits our ability to conduct a thorough analysis of their impact. Consequently, the efficacy of theory-driven interventions in this domain remains uncertain. While the theory may not necessarily result in a favorable impact on outcomes supporting the intervention, it assists in identifying, among a multitude of options, the intervention components that could prove effective, further supporting the development of interventions in future research.^78^

### Strengths and limitations

To the best of our knowledge, this review is the first to present an inclusive summary of the impact of pharmacist intervention in perioperative settings on various clinically relevant outcomes. The study protocol was preregistered on PROSPERO. Data extraction was performed by 4 researchers utilizing the DEPICT-2 tool, ensuring a consistent and unbiased approach.^26^ Through adherence to PRISMA guidelines, the systematic review’s reporting was meticulously executed.

This review has certain limitations. Firstly, there was a language restriction to Arabic and English, potentially excluding relevant literature in other languages. Secondly, the outcomes of the review exhibit heterogeneity; similar clinical outcomes were assessed in diverse ways, precluding the derivation of a definitive statistical conclusion. Conflicting findings were observed in some outcomes, particularly in studies examining the perioperative management of chronic conditions like hypertension and diabetes and other conditions that require close monitoring, which could be attributed to the fact that the studies included were low in quality, were small in number, and were not targeted to studying those specific outcomes. Thirdly, a notable limitation is the small sample size in many of the reviewed studies, indicating insufficient statistical power to establish the impact of pharmacist intervention on the outcomes of interest. Fourth, the brief follow-up periods in numerous studies, often confined to the admission period, presented a challenge. Lastly, the generalizability of our findings is limited due to the predominant inclusion of studies from China and the United States.

### Future directions

A substantial void in the existing literature necessitates investigating the influence of pharmacist interventions on critical perioperative outcomes by focusing on factors such as elevated blood glucose, hypertension, and perioperative bleeding, all of which have been demonstrated to contribute to complications, procedural delays, and other unfavorable outcomes when left unregulated.^79,80^ Emerging studies should construct pharmacist interventions grounded in theoretical frameworks, taking into account the distinctive characteristics of surgical settings and the workflow of surgeons.^81,82^ We urge subsequent research to furnish comprehensive descriptions of interventions, covering structures, processes, and outcomes to enhance reproducibility, with the additional recommendation of employing the DEPICT-2 tool. Furthermore, there is a need for additional exploration into the implications of pharmacist prescribing in clinical pharmacy practice, given its potential benefits, including accelerated access to medications and alleviation of physician workload. Finally, it is imperative to establish a standardized guideline instructing pharmacists in the perioperative management of patients. This guideline should be evidence-based and address the issues commonly encountered by pharmacists in their evolving responsibilities within surgical settings. While previous guidelines, such as those from ASHP,^19^ have been published, there is a need for guidelines that meet the required level of detail to effectively guide surgical pharmacists in navigating the extremely wide range of intricate decisions they confront on a daily basis.

## Conclusion

There is some evidence for the positive impact of pharmacist intervention on clinical outcomes and therapeutic optimization in perioperative settings; however, this evidence is of low quality and insufficient volume. Positive impacts were noted in findings related to pain control, VTE prophylaxis, nausea and vomiting, and surgery-related stress ulcers. Nonetheless, there is inconsistency in the findings related to medication management (ie, the ability to achieve desired therapeutic ranges) and management of chronic conditions (hypertension and type 2 diabetes). There are a variety of areas in which the pharmacist could potentially play significant roles that have not been investigated in the current body of literature, such as anticoagulation management or asthma care during the perioperative period. Future well-designed randomized trials with larger sample size and appropriate outcome measurements should be initiated.

## Data Availability

The data underlying this article will be shared on reasonable request to the corresponding author.

## Additional information

The authors provided the following information on contributions to their work: L.N. contributed to the study design, systematic review planning, data extraction and interpretation of data, and data analysis and led the article screening, article selection, and manuscript writing. S.K. contributed to the study design, data extraction and interpretation of data, article screening, article selection, and revision of the manuscript. B.A. and M.A. were involved in article selection, data extraction, manuscript writing, and revision of the manuscript.

## Appendix A—Results of quality assessment of included studies using Crowe Critical Appraisal Tool

**Table:**

Source	Prelim- inaries	Intro- duction	Design	Sampling	Data collection	Ethical matters	Results	Discussion	Final score	Total, %^a^	Final quality rating
Alsheikh et al (2021)^41^	4	4	3	2	2	4	4	4	27	67.5	Low
Barat et al (2023)^30^	4	3	2	4	4	5	3	4	29	72.5	Low
Cronin et al (2009)^37^	4	3	4	2	2	3	3	4	25	62.5	Low
Decelis et al (2015)^31^	4	4	3	2	3	3	2	3	24	60	Low
Fitzpatrick et al (2023)^32^	5	5	2	1	4	5	4	5	31	77.5	Moderate
Hale et al (2013)^38^	4	4	3	4	4	3	4	4	30	75	Moderate
Katada et al (2017)^42^	4	4	3	3	4	3	4	4	29	72.5	Moderate
Kiraci et al (2023)^39^	4	3	2	4	3	4	2	3	25	62.5	Low
Luo et al (2017)^45^	4	4	4	3	4	4	5	4	32	80	Moderate
McPhee et al (1991)^33^	3	3	1	2	2	1	2	2	16	40	Low
Patel et al (2022)^34^	4	4	3	3	3	3	4	3	27	67.5	Low
Shang et al (2021)^40^	4	3	3	4	4	1	4	4	27	67.5	Low
Smith et al (2018)^35^	4	4	3	3	4	5	4	4	31	77.5	Moderate
Smith et al (2023)^43^	4	4	2	5	4	4	3	3	29	72.5	Moderate
Tong et al (2022)^47^	5	4	4	2	2	5	3	4	29	72.5	Moderate
Wang et al (2019)^48^	2	3	3	1	3	3	3	3	21	52.5	Low
Wang et al (2020)^46^	4	4	3	4	4	3	4	5	31	77.5	Moderate
Wang et al (2023)^36^	3	4	3	3	3	5	5	4	30	75	Moderate
Yang et al (2019)^44^	4	4	3	1	2	3	4	4	30	75	Moderate

a Percentage of quality standards met.

## Appendix B—Characteristics of interventions performed by pharmacists (per DEPICT-2 assessment)

**Table:**

Source	Pharmacist intervention	Recipient	Intervention	Mode of contact	Methods of communication	Settings	Timing of pharmacy contact and frequency	Clinical data sources	Source guide for intervention	Materials that supported the action
**Focus of intervention: pain control and opioid consumption**										
Barat et al (2023)^30^	Pharmacy consultation: general open-ended questions to determine outpatients’ existing knowledge, beliefs, or apprehensions of pain medication, followed by second step (individualized presentation of prescription and methods for taking pain medication, recommendations on optimal times for taking pain medication, and a presentation on drug interactions and possible adverse effects and actions to be taken in case of adverse effects) Follow-up, contact with community pharmacist to share update	Patients	Analgesia	One-on-one (patient)	Face-to-face	Ambulatory/primary care setting	Several days before surgery, after discharge	Patient report, analgesia prescription	NR	Information summary sheet
Decelis et al (2015)^31^	Patients received pharmaceutical care plan, including face-to-face explanation and educational materials about pain causes and management; this included a leaflet with pictures explaining pain origins and control methods along with a 6-week chart detailing daily paracetamol doses	Patients	Analgesia (paracetamol)	One-on-one	Face-to-face	Hospital (bedside and outpatient clinic)	Postoperative, with follow-up at 2, 4, and 6 weeks	Pain assessment questionnaire	Primary literature	None
Fitzpatrick et al (2023)^32^	Review of electronic notes and phone call with patients to confirm history and demographics and answer patients’ questions Discussion with surgical team to highlight or resolve perioperative medical issues Individualized discharge prescription written, emailed, dispensed, and supplied to wards before admission	Patients	None	One-on-one (patient), contact with group (surgical team)	Face-to-face, telephone, written	Recipient’s home	1-2 weeks before admission, 7-10 days post discharge	NHS virtual private network, ARISE dataset	Evidence-based guideline produced in collaboration with surgical MDT, health board guidance for VTE risk assessment and procedures	None
McPhee et al (1991)^33^	Phase 1: guidelines on use of postoperative IM narcotics developed by pharmacy department, with input from 2 physicians and 1 nurse, and distributed Phase 2: clinical pharmacist promoted optimal use of narcotics and presented 1 in-service educational program 8 times for nurses; involved physicians either attended in-service session or had one-on-one review of guidelines with the pharmacist; attending staff physicians heard clinical pharmacist advocate use of guidelines at teaching rounds; also, participation in ward rounds and reinforcement of guidelines	Physicians and nurses	Narcotics	Phase 1, NA; phase 2, contact with group with nurses, contact with group or one-on-one with physicians	Phase 1, mail; phase 2, group or one-on-one sessions	Hospital	At beginning of each phase	NR	NR	None
Patel et al (2022)^34^	Consensus-gathering process to improve multimodal analgesia, reduce high-risk drug combinations, and lower opioid exposure Education provided to nursing staff, nursing supervisors, and orthopedics residents	Medical staff (resident physicians) and nurses	Opioids	One-on-one, contact with group	Face-to-face	Hospital bedside	NR	EMR	Best available evidence for multimodal postoperatve analgesia and safe opioid use	None
Smith et al (2018)^35^	Mailed brochure (10 days before surgery) described what patients should expect regarding opioid use and pain control after surgery Mailed brochure (15 days after THA/TKA) explained opioid use topics, including rationale for opioid use after surgery, opioid tapering expectations after surgery, and potential adverse effects of opioids Follow-up telephone call from pharmacist who used motivational enhancement principles to reinforce information in the brochure if patients filled a prescription for opioid in 28-90 days after surgery	Patients	Opioids	One-on-one (if needed)	Brochure, telephone (if needed)	Recipient’s home	One time 10 days before admission, one time 15 days post discharge, follow-up phone call 28 days post discharge (if needed)	EMR	Brochure developed by research team using qualitative methods, with input from patients and orthopedic clinicians; telephone calls used motivational enhancement principles	Brochure
Wang et al (2023)^36^	Preadmission assessment, with preoperative intervention for high-risk patients Postoperative (monitoring and analgesia adjustment, MDT rounds, case discussions, use of WeChat workgroups to communicate with patients, ADR monitoring) Discharge review and management	Patients, medical staff	Analgesia	One-on-one (patient), contact with group (surgical team)	Face-to-face, written	Hospital bedside	Within 48 hours of admission and within 24 hours after pre-admission assessment; 24 hours post-operatively and 24-48 hours before discharge	EMR	EUSEM guideline, ERAS protocol, expert opinions	Brochures
**Focus of intervention: VTE prophylaxis**										
Cronin et al (2009)^37^	Thromboprophylaxis risk factor assessment and prescriber order sheet for venous thromboprophylaxis for all patients Clinical pharmacist provided education to team about published guidelines, risk factor assessment and prescriber order sheet, and protocol changes for prophylaxis and conducted daily chart reviews and twice-weekly patient care rounds with orthopedic team	Patients	VTE prophylaxis	Contact with group	Face-to-face; thromboprophylaxis team, patient care rounds	Hospital	Continuously during patient admission	Medical records	ACCP guidelines	VTE prophylaxis risk factor assessment and prescriber order sheet
Hale et al (2013)^38^	Usual pharmacy care in addition to prescribing (continuing, discontinuing, and initiating medications, with countersignature by physician)	Patients and physicians	None	One-on-one	Face-to-face	Hospital (bedside and outpatient clinic)	Preoperative	Medication chart	Clinical guidelines and hospital VTE prophylaxis guidelines	None
Kiracı et al (2023)^39^	Educational intervention: online information (on analysis of surgeons’ anonymized current practices) and education (on Caprini RAM, VTE risks, indications, choice, doses, frequency and duration of thromboprophylaxis) provided to all surgeons, and posters summarizing education posted in visible areas in all general surgery wards Clinical pharmacy services: assessment of VTE risk and appropriateness of VTE prophylaxis, with recommendations provided	Surgeons	VTE prophylaxis	NR	Not detailed; however, information was available online for surgeons in first phase	Hospital	NR	NR	Caprini risk assessment model (2013) and ACCP guideline (2012)	Online information, poster
Shang et al (2021)^40^	Conducting thrombosis and bleeding risk assessment for patients, then consulting with physicians to formulate antithrombotic treatment protocols Optimizing perioperative medication regimens and discharge prescriptions Providing consultation for medical staff and patients throughout hospital stay Providing anticoagulant-related training for medical staff every quarter Following up on thrombosis after TJA in first and third month after surgery	Patients, medical staff	Anticoagulants	Contact with group (medical staff and patients)	Face-to-face	Hospital bedside	Continuously during patient admission, after discharge follow-ups	EMR	Guidelines of ACCP and orthopedic branch of Chinese Medical Association, drug instructions	None
**Focus of intervention: postoperative acute and chronic medication management**										
Alsheikh et al (2021)^41^	Pre–kidney transplant evaluation and daily rounds to monitor therapy and answer questions of the clinical team Daily meetings with transplant MDT to discuss therapeutic plan Patient medication education during hospital stay and on discharge Follow-up of newly transplanted patients in outpatient clinic on weekly basis Recommendation of dose adjustments for several medications based on changes in creatinine clearance	Patients and physicians	All medications, with focus on immunosuppressants	One-on-one	Face-to-face, telephone	Hospital (bedside and outpatient clinic)	Continuously during patient admission	Medication chart/patient history taking	Clinical guidelines of CMS	None
Katada et al (2017)^42^	Pharmacists evaluated and revised daily warfarin doses based on INR and managed timing of blood sampling accordingly	Physicians	Warfarin	One-on-one	Face-to-face	Hospital	Daily contact post-operatively	NR	Clinical guidelines, hospital protocol	None
Smith et al (2023)^43^	Use of clinical guidance tool for glycemic management in well-controlled subset of patients	Patients	Antidiabetic medications	Contact with group (surgical team and patients)	Face-to-face	Hospital bedside	Continuously during patient admission	EMR	Local glycemic management model at Mayo Clinic	None
Yang et al (2019)^44^	Direct patient care and medication management during hospitalization Reviewing medication regimens and resolving medication‐related problems Medication reconciliation Answering drug information questions Therapeutic drug monitoring Making therapeutic recommendations Patient education	Patients	None	One-on-one (patient), contact with group (medical team)	Face-to-face	Hospital bedside	Continuously during patient admission	NR	NR	None
**Focus of intervention: surgery-related stress ulcer and nausea/vomiting prophylaxis**										
Luo et al (2017)^45^	Provide educational sessions and handouts about SUP for medical teams Collect information about the patient from the EMR and health information system Judge appropriateness of use of prophylactic acid suppressants, then immediately communicate recommendation, if any, to prescriber Report to the hospital administration every week	Physicians	PPIs and H_2_ blockers	One-on-one	Face-to-face	Hospital	Continuously during patient admission	EMR	Hospital protocol	None
Wang et al (2020)^46^	Review of patients’ medical records Feedback on current PONV prophylaxis provided to surgical team, with discussion of evidence Strategies for PONV prophyolaxis optimization highlighted	Patients, surgeons	Antiemetics	Contact with group (surgeons, anesthesiologists, patients)	Face-to-face, written	Hospital bedside	Continuously during patient admission	EMR	ASA and hospital protocol	None
**Focus of intervention: total parenteral nutrition**										
Tong et al (2022)^47^	Development and implementation of evaluation standard	Medical staff	TPN	No direct contact (patient), one-on-one (doctors)	Face-to-face, written	Hospital bedside	NA	EMR	ESPEN, ASPEN, CSPEN	None
Wang et al (2019)^48^	Development of software for pharmacist-led TPN management	Patients	TPN	N/A	N/A	Hospital	NA	EMR	NR	None

Abbreviations: AACP, American Association of Chest Physicians, ADR, adverse drug reaction; ARISE, Arthroplasty Rehabilitation in Scotland Endeavour; ASA, American Society of Anesthesiologists; ASPEN, American Society for Parenteral and Enteral Nutrition; CMS, Centers for Medicare and Medicaid Services; CSPEN, Chinese Medical Association for Parenteral and Enteral Nutrition; EMR, electronic medical record; ESPEN, European Society for Clinical Nutrition and Metabolism; EUSEM, European Society for Emergency Medicine; H_2_, histamine type 2 receptor; INR, international normalized ratio; MDT, multidisciplinary team; NA, not applicable; NR, not reported; PONV, postoperative nausea and vomiting; PPI, proton pump inhibitor; TJA, total joint arthroplasty; TPN, total parenteral nutrition; VTE, venous thromboembolism.
